# The Beneficial Effect of the First COVID-19 Lockdown on Undergraduate Students of Education: Prospective Cohort Study

**DOI:** 10.2196/27286

**Published:** 2022-02-23

**Authors:** Gili Joseph, Hadas Schori

**Affiliations:** 1 Department of Physical Activity and Movement Faculty of Science Seminar Hakibbutzim College of Education Tel Aviv Israel

**Keywords:** sleep quality, exercise, well-being, undergraduate students, COVID-19 lockdown, COVID-19

## Abstract

**Background:**

The COVID-19 pandemic has been spreading consistently since the beginning of 2020. On February 27, 2020, the first patient with coronavirus was diagnosed in Israel. On March 14, 2020, the Israeli government declared a general lockdown that lasted about a month, which altered the lives of the entire population.

**Objective:**

The objective of this paper is to evaluate the change in the well-being, physical activity, and sleep quality of undergraduate students of education at 2 time points: before (November 2019) and during (April 2020) the first COVID-19 lockdown.

**Methods:**

In total, 533 undergraduate students of education submitted an online questionnaire before the lockdown and at its end. The questionnaire comprised 4 parts: a (1) sociodemographic and (2) weekly exercise questionnaire taken from the International Physical Activity Questionnaire–Short Form; (3) sleep quality, rated using the Mini Sleep Questionnaire; and (4) well-being, rated using the short version of the Mental Health Inventory. This was a pre-post prospective cohort questionnaire study.

**Results:**

It was predicted that there would be a decrease in the aforementioned parameters. Contrary to all expectations, an increase was observed in all 3. Results showed that during the lockdown, there was an increase in the level of exercise students engaged in. Overall, 102 (61.4%) of 166 students engaged in a greater amount of physical activity during the COVID-19 lockdown compared to 150 (40.9%) of 367 students who engaged in a greater amount of physical activity before COVID-19. Levels of sleep quality (mean 5.34 [SD 0.92] vs mean 5.12 [SD 0.46], *P*=.02) and well-being (mean 3.79 [SD 0.62] vs mean 3.67 [SD 0.59], *P*=.02) were also higher during the COVID-19 lockdown.

**Conclusions:**

These findings indicate that undergraduate students seem to have taken advantage of the change in lifestyle due to the lockdown, directing the free time toward improving health by engaging in more physical activity, thus improving sleep quality and well-being.

## Introduction

The year 2020 challenged the entire world. Coronavirus (COVID-19) was declared a global epidemic by the World Health Organization [1]. This led many countries to take various precautions to prevent the spread of the virus. The severity of the general closure varied between countries, each implementing different measures ranging from increased enforcement of limitations (limited unessential social interaction, limited mobility, limited work-related activities) up to a general lockdown [2]. On March 14, 2020, the Israeli government declared a general lockdown, which included restricting mobility, reducing working capacity by 10%-20%, and closing the doors of the entire educational system for all ages [3], lasting approximately 2 months.

The lockdown, as well as many other limitations, had a serious impact on our lifestyle. Studies have shown an increase in depression and self-reported stress and a reduction in well-being during the first COVID-19 lockdown [4-7]. In addition, a reduction in the amount of physical activity performed was found among different populations worldwide; this has had a negative effect on the well-being of the population [8-10]. Furthermore, a decrease in sleep quality was found [11,12].

Sleep is 1 of the essential components of health [13]. Poor sleep quality may adversely affect the immune system, learning abilities, blood pressure, psychological status, and more [14-16]. In general, any changes in our lifestyle may affect our sleep quality [17,18]. In addition, there was a major, sudden transformation in our lifestyle, as imposed upon the population during the first COVID-19 lockdown. The lockdown not only affected sleep quality but also caused sleeplessness [19-22].

Exercise is 1 of the most recommended ways to improve an individual's health [23]. Physical activity can improve sleep quality [24], and regular exercise can be 1 of the methods of treating people with sleep disorders [25,26]. Moreover, studies have shown small-to-moderate beneficial effects of regular exercise on total sleep time, sleep efficiency, sleep onset latency, and sleep quality [25,27]. Acute and long-term exercise has been documented in a number of studies as 1 of the factors that can increase slow-wave sleep and total sleep time, as well as decreasing the period between the initial attempt to fall asleep and the onset of sleep [28-30]. In addition, aerobic physical activity was shown to improve sleep quality for people with sleep disorders [31].

Physical activity is also considered 1 of the factors that can help people improve their well-being; it is often recommended as a way to deal with stress [32]. People with a variety of mental disorders have shown improvement in their mental condition when implementing physical activity in their daily routine [33-35].

Regarding undergraduate students, there are some findings that present a decrease in physical activity as well as well-being during the first COVID-19 lockdown, due to the transition to remote teaching and a major change in their lifestyle [36-38]. However, research conducted on students studying health and science revealed that during the COVID-19 lockdown, they spent more time engaging in physical activity but also increased the amount of time they were sedentary [39].

This study aims to examine the effect of the first COVID-19 lockdown on undergraduate Israeli students of education. These students are characterized by a nonsedentary lifestyle due to their mandatory practical teaching. During the first COVID-19 lockdown, all practical lessons were transferred to remote teaching and therefore students were forced to increase their sitting time. Here, we aim to analyze their physical activity, well-being, and sleep quality before and during the COVID-19 lockdown.

## Methods

### Subjects

Subjects comprised over 600 students from a college of education in the center of Israel. The questionnaire was distributed to first-, second-, and third-year students in the science and education faculties. The students who replied were included in the study (N=367), while the students who did not reply were excluded (N=233). All 367 (100%) students replied to the pre-COVID-19 questionnaire, and 166 (45.2%) students replied to the questionnaire during the COVID-19 lockdown (postquestionnaire). Altogether, 533 questionnaires were submitted. Ordinarily, during the 4 years of the students' studies, they attend the college on campus at least 3 days a week, and they are obligated to teach at least once a week in a proscribed school during all 4 of their college study years. In their fourth year, they attend the college only once a week, and during the rest of the week, they teach in schools, under the supervision and guidance of experienced and expert teachers from those schools. This study received ethical approval from the ethics committee of the Seminar Hakibutzim College, Tel Aviv, Israel.

### Research Tools

The questionnaire was submitted by the students using Google Forms. The questionnaire included 4 sections: sociodemographic questions, questions pertaining to the level and intensity of their weekly exercise, a well-being questionnaire, and a questionnaire regarding their quality of sleep. The Hebrew version of the exercise questionnaire was taken from the International Physical Activity Questionnaire–Short Form (IPAQ-SF) [40]. The students were asked to note how many times and the number of hours they exercise per week and the number of times and hours a week they engage in physical activity such that it causes sweat production and strenuous breathing. The questions were based on a Likert scale of 1-7, where 1 represents never and 7 represents always. The 4 exercise questions were grouped into 1 variable, and then a dichotomous variable was rebuilt and the population was divided into 2 groups by the median of 3.5. Students with a score of 0-3.49 in exercise were categorized as engaging in a lesser amount of exercise. Students with a score of 3.5-7 in exercise were categorized as engaging in a greater amount of exercise. The well-being questionnaire used was the short version of the Mental Health Inventory (MHI) developed by Veit and Ware [41] and validated by Florian and Drori [42] and included 10 questions: Cronbach α=.96. Sleep quality was measured using the Mini Sleep Questionnaire (MSQ), which included 10 questions that relate to quality of sleep using a Likert scale of 1-7 [43].

### Research Process

During the second and third weeks of the first semester (November 2019), the researchers entered the classrooms and explained the objectives of the study. Immediately following that, the questionnaire was distributed, and the students submitted it using Google Forms. The second questionnaire was distributed via email because at that time (at the end of the first COVID-19 lockdown, April 2020), the students studied only online and did not study on campus. Since the students answered the questionnaire of their own volition, and the questionnaire was anonymous, not all of them chose to participate; thus, only 166 (45.2%) of 367 students submitted the second questionnaire. In the second questionnaire, the students were asked to answer the questions with reference to the COVID-19 lockdown period.

### Statistical Analysis

An independent *t* test analysis was performed to measure the difference between the amount of exercise, sleep quality, and well-being among undergraduate students of education before and during the COVID-19 lockdown, and the difference between well-being and the quality of sleep by the amount of exercise engaged in by the students. The analysis of the answers to the questionnaires before and during the COVID-19 lockdown were not matched; thus, an independent *t* test analysis was performed. A chi-square analysis was performed to measure the difference in the amount of exercise before and during the COVID-19 lockdown. A 2-way ANOVA was conducted in order to find the main effects and interactions between the period when the questionnaire was submitted (before or during the COVID-19 lockdown), the year of study/field of study/gender, and the 3 dependent variables: amount of exercise, sleep quality, and well-being. Results were statistically significant at the .05 significance level.

## Results

### Participant Characteristics

The students who participated in this study are undergraduate students of education from the faculties of science and education. The average age of the students was 25.3 (SD 4.5) years. Most of the students were female (291/367, 79.3%) and single (304/367, 82.8%). In addition, 163 (44.4%) of the students were in their first year of studies and 204 (55.6%) in their second and third years of studies. Furthermore, 130 (35.4%) of the students studied in the Department of Physical Education, while the rest studied in other education disciplines, such as science, elementary school, early childhood education, and special education. All 367 (100%) students submitted the first questionnaire, while 166 (45.2%) students submitted the second one (Table 1). There was no statistical difference between the students answering the prequestionnaire and those who answered the postquestionnaire concerning their characteristics.

**Figure 1:**
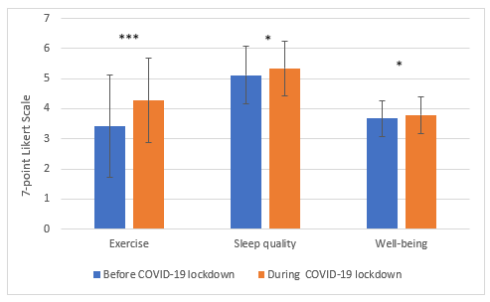
The difference between exercise, sleep quality and well-being before and during the COVID-19 lockdown. Independent *t* test analysis was engaged in to measure the difference between the amount of exercise, sleep quality and well-being among undergraduate students of Education. N=367 - pre-COVID-19, N=166 - during COVID-19 lockdown, (***P<.001, * *P*<.05).

The 3 main research questions asked were whether there was any difference between the 2 periods (1) in the amount of exercise engaged in, (2) in their well-being, and (3) in their quality of sleep. An independent *t* test analysis was conducted, and the results showed that the students engaged in more exercise during the lockdown compared to the beginning of the same school year (mean 4.27 [SD 1.4] vs mean 3.4 [SD 1.7], *P*<.001). They also had better quality of sleep (mean 5.34 [SD 0.92] vs mean 5.12 [SD 0.46], *P*=.02) and better well-being (mean 3.79 [SD 0.62] vs mean 3.67 [SD 0.59], *P*=.02); see Figure 1.

**Table 1:** Descriptive characteristics of the participants.

Characteristics		Prequestionnaire (N=367)	Postquestionnaire (N=166)
**Age (years), mean (SD)**		25.3 (4.5)	25.6 (1.5)
**Gender, n (%)**			
	Female	291 (79.3)	122 (73.5)
	Male	76 (20.7)	44 (26.5)
**Marital status, n (%)**			
	Single	304 (82.8)	130 (78.3)
	Other status	63 (17.2)	36 (21.7)
**Field of study, n (%)**			
	Physical education	130 (35.4)	101 (60.8)
	Other study fields	237 (64.6)	65 (39.2)
**Study year, n (%)**			
	First year	163 (44.4)	64 (38.6)
	Second and third years	204 (55.6)	102 (61.4)

When analyzing the amount of exercise the students engaged in before and during the COVID-19 lockdown, 150 (40.9%) of the 367 students engaged in a greater amount of exercise before the COVID-19 lockdown. However, during the COVID-19 lockdown, the number of students engaging in a greater amount of exercise increased to 102 (61.4%) of 166 students (Table 2). The chi-square analysis showed a significant difference (*χ*^2^_1_=19.41, *P*<.001). The φ correlation showed low (0.2) but significant (*P*<.001) strength. An independent *t* test analysis was performed to compare the quality of sleep among the students who engaged in a greater amount of exercise compared to those who engaged in a lesser amount of exercise during the COVID-19 lockdown. Those who engaged in a greater amount of exercise had better sleep quality (mean 5.57 [SD 0.84] vs mean 4.96 [SD 0.93], *P*<.001). No difference was found in their well-being (Table 3).

A 2-way ANOVA was conducted to compare the main effects of the interaction of the time when the questionnaire was submitted (before or during the COVID-19 lockdown) and the year of study/field of study/gender on the amount of exercise, sleep quality, and well-being (Tables 4-6).

As shown in Table 4, the effect of the 2 independent variables (time when the questionnaire was submitted and the year of study) on the amount of exercise, sleep quality, and well-being was studied. Results showed effects that were statistically significant at the 0.05 significance level only for the time when the questionnaire was submitted. Regarding the amount of exercise, the main effect of the time when the questionnaire was submitted yielded an F_(1, 527)_ ratio of 33.0 (*P*<.001), indicating a significant difference between the amount of exercise the students engaged in before the COVID-19 lockdown (mean 3.4 [SD 1.7]) and during the COVID-19 lockdown (mean 4.28 [SD 1.46]). No effect was found of the year of study, and no interaction was found between the time when the questionnaire was submitted and the year of study. When examining the quality of sleep, the main effect of the time when the questionnaire was submitted yielded an F_(1, 527)_ ratio of 6.17 (*P*=.01), indicating a significant difference between the students’ quality of sleep before the COVID-19 lockdown (mean 5.12 [SD 0.96]) and during the COVID-19 lockdown (mean 5.35 [SD 0.91]). No effect was found of the year of study, and no interaction was found between the time when the questionnaire was submitted and the year of study. The same trend was found regarding well-being. The main effect of the time when the questionnaire was submitted yielded an F_(1, 527)_ ratio of 5.79 (*P*=.02), indicating a significant difference between the students’ well-being before the COVID-19 lockdown (mean 3.67 [SD 0.59]) and during the COVID-19 lockdown (mean 3.8 [SD 0.61]). No effect was found of the year of study, and no interaction was found between the time when the questionnaire was submitted and the year of study.

**Table 2:** The number and percentage of students engaging in a lesser and greater amount of exercise before and during the COVID-19 lockdown.

Period	Students engaging in a lesser amount of exercise, n (%)	Students engaging in a greater amount of exercise, n (%)	*P* value
Before COVID-19 lockdown (N=367)	217 (59.1)	150 (40.9)	<.001
During COVID-19 lockdown (N=166)	64 (38.6)	102 (61.4)	<.001

**Table 3:** The difference in well-being and sleep quality in reference to the amount of exercise engaged in by the students during the COVID-19 lockdown (N=166).

Amount of exercise		n (%)	Mean (SD)	*P* value
**Well-being**				
	Lesser amount of exercise	64 (38.5)	3.7 (0.73)	.07
	Greater amount of exercise	102 (61.5)	3.9 (0.53)	.07
**Sleep quality**				
	Lesser amount of exercise	64 (38.5)	4.96 (0.93)	<.001
	Greater amount of exercise	102 (61.5)	5.6 (0.84)	<.001

**Table 4:** Results of 2-way ANOVA to compare the main effects of the independent variables “time when the questionnaire was submitted” and “year of study” and their interaction on the amount of exercise, sleep quality, and well-being.

Variables		F_1_ ratio	*P* value
**Dependent variable: amount of exercise**			
	Time when the questionnaire was submitted	33.0	<.001
	Year of study	0.29	.59
	Interaction	0.79	.37
**Dependent variable: sleep quality**			
	Time when the questionnaire was submitted	6.17	.01
	Year of study	1.54	.22
	Interaction	0.028	.87
**Dependent variable: well-being**			
	Time when the questionnaire was submitted	5.79	.02
	Year of study	0.43	.51
	Interaction	0.02	.89

**Table 5:** Results of 2-way ANOVA to compare the main effects of the independent variables “time when the questionnaire was submitted” and “field of study”^a^ and their interaction on the amount of exercise, sleep quality, and well-being.

Variables		F_1_ ratio	*P* value
**Dependent variable: amount of exercise**			
	Time when the questionnaire was submitted	9.798	.002
	Field of study	166.29	<.001
	Interaction	8.34	.004
**Dependent variable: sleep quality**			
	Time when the questionnaire was submitted	2.403	.12
	Field of study	11.363	<.001
	Interaction	0.435	=.51
**Dependent variable: well-being**			
	Time when the questionnaire was submitted	4.81	.03
	Field of study	0.010	.92
	Interaction	0.003	.96

^a^Field of study is a comparison between physical education students compared to students form all the other fields together.

**Table 6:** Results of 2-way ANOVA to compare the main effects of the independent variables “time when the questionnaire was submitted” and “gender” and their interaction on the amount of exercise, sleep quality, and well-being.

Variables		F_1_ ratio	*P* value
**Dependent variable: amount of exercise**			
	Time when the questionnaire was submitted	8.3	.004
	Gender	46.3	<.001
	Interaction	7.38	.01
**Dependent variable: sleep quality**			
	Time when the questionnaire was submitted	1.18	.04
	Gender	4.09	.27
	Interaction	2.018	.16
**Dependent variable: well-being**			
	Time when the questionnaire was submitted	5.29	.02
	Gender	2.94	.09
	Interaction	0.155	.69

**Figure 2:**
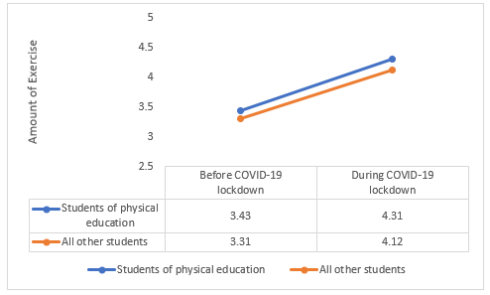
Interaction effect between the time of submitting the questionnaire (before or during the COVID-19 lockdown) and the field of study on the amount of exercise (2-way ANOVA). *P*<.05.

Table 5 presents the results of a 2-way ANOVA when the 2 independent variables this time were the time when the questionnaire was submitted and the field of study. The 2-way ANOVA showed effects on the amount of exercise that were statistically significant (*P*<.05). The main effect of the time when the questionnaire was submitted yielded an F_(1, 529)_ ratio of 9.798 (*P*=.002), indicating a significant difference between the amount of exercise the students engaged in before the COVID-19 lockdown (mean 3.4 [SD 1.7]) and during the COVID-19 lockdown (mean 4.28 [SD 1.46]). The main effect of the field of study yielded an F_(1, 529)_ ratio of 166.29 (*P*<.001), indicating a significant difference between the amount of exercise the physical education students engaged in (mean 4.77 [SD 1.3]) and the students from all the other fields of study (mean 2.85 [SD 1.46]). The interaction effect between the time when the questionnaire was submitted and the field of study was significant (F_(1, 529)_=8.34, *P*=.004), showing an ordinal interaction between the variables, meaning a greater amount of exercise was performed during the COVID-19 lockdown and among the students of physical education (Figure 2). Regarding sleep quality, the main effect of the field of study showed effects that were statistically significant at the 0.05 significance level and yielded an F_(1, 529)_ ratio of =11.363 (*P*=.001), indicating a significant difference between the sleep quality of students of physical education (mean 5.37 [SD 0.87]) compared to students from all the other disciplines (mean 5.05 [SD 0.99]). Regarding sleep quality, no interaction was found between the time when the questionnaire was submitted and the field of study (F_(1,529)_=0.435, *P*=.51).

**Figure 3:**
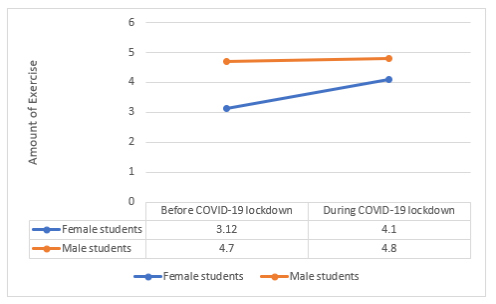
Interaction effect between the time of submitting the questionnaire (before or during the COVID-19 lockdown) and gender on the amount of exercise (2-way ANOVA). Before COVID-19 lockdown: female students, n=367; male students, n=166. During COVID-19 lockdown: female students, n=424; male students, n=109. *P*<.05.

A 2-way ANOVA was conducted for the influence of another pair of independent variables: time when the questionnaire was submitted and gender (Table 6). Regarding the amount of exercise, the main effect of the time when the questionnaire was submitted yielded an F_(1, 529)_ ratio of 8.3 (*P*=.004), indicating a significant difference between the amount of exercise the students engaged in before the COVID-19 lockdown (mean 3.4 [SD 1.7]) and during the COVID-19 lockdown (mean 4.28 [SD 1.46]). The main effect of gender yielded an F_(1, 529)_ ratio of 46.3 (*P*<.001), indicating a significant difference between the amount of exercise the male students engaged in (mean 4.77 [SD 1.49]) compared to female students (mean 3.4 [SD 1.49]). The interaction effect between the time when the questionnaire was submitted and gender was significant (F_(1, 529)_=7.38, *P*=.01), showing an ordinal interaction between the variables, meaning a greater amount of exercise was performed during the COVID-19 lockdown among male students, although it is interesting to observe that female students showed a more significant change in the amount of exercise during the COVID-19 lockdown (Figure 3 and Table 6).

## Discussion

### Principal Findings

Undergraduate students of education showed improvement in their well-being, sleep quality, and exercise during the first COVID-19 lockdown. These results were contrary to all expectations, since the restriction enforced due to the COVID-19 pandemic was shown to have a serious impact on the population, resulting in an enormous change in lifestyle and in daily routine [44,45]. Moreover, some studies have shown that during the first COVID-19 lockdown or during quarantine, there was a decrease in well-being, sleep quality, and physical activity among most of the general population [5,7,22,46-51]. Our results can be explained by other studies that have shown that the more time people have, the more they invest in their hobbies and in exercising [52]. A study conducted on European Union citizens revealed that 73.1% exercised in their free time [53], and a study in Spain showed that more than 50% enjoyed new positive experiences during the lockdown; specifically, students of health and science reported an increase in both the number of days in which they engaged in physical activity and in the total number of minutes of physical activity they engaged in per week [39].

However, there are studies showing that during the COVID-19 lockdown, people experienced more time alone, increased the use of electronic devices, and stayed indoors more, which all led to a reduction in sleep quality and a decrease in their well-being [4,11,19-22,50,54,55]. This study does not contradict these findings but complements them with additional aspects of life that have not been tested yet, such as the possible benefit of physical activity on well-being and sleep quality in a time when lifestyle changes may cause sudden distress. The students who participated in this study are studying toward earning a teaching certificate in an Israeli education college in Tel Aviv. Typically, they commute between 1 and 3 hours every day to get to college (the college has no dorms) or to the schools where they teach. After school hours, many of them need to go out to work to support themselves. The first COVID-19 lockdown forced the students to stay at home; discontinue working, teaching in the schools, and studying on campus; and switch to distance learning or distance teaching. Although this situation increased many sitting hours, it also led to more free hours because of less travel time and less or no work (88% reported working less than normal times; see Multimedia Appendix 1). The statistical correlation found between exercising more and sleeping better might be explained by the free time the students gained because of less or no work and no commuting, while not having additional personal or family responsibilities. Doing more exercise and sleeping better may have improved their well-being. These results are in line with previous works showing that free-time activities play an important role in subjective well-being [56] and that high levels of free-time exercise are associated with benefits for psychological well-being [57].

Studies have demonstrated that physical activity can improve well-being regardless of gender, socioeconomic status, and health status [8,58-61]. In addition, physical activity (especially high levels) can improve sleep quality [24,25,62,63]. Furthermore, studies have shown that the amount and extent of physical activity are positively associated with the researched population's well-being. During the COVID-19 lockdown period, the student population was at high risk for developing mental health problems, indicating a decrease in their well-being [48,49]. Here, it was shown for the first time that there is not only a correlation between an increase in the intensity and the amount of the students' exercise (102/166 [61.4%] of the students engaged in a greater amount of physical activity) but also a correlation between the students who engaged in a greater amount of exercise and their well-being and sleep quality.

Transitional times in life, such as going to college and being a freshman, include leaving home, gaining more independence, and adhering to less structured schedules, and other stressful conditions can affect sleep patterns and well-being [64-66]. Thus, there was expected to be a difference between first-year and second- and third-year students with regard to maintaining a healthy lifestyle. Here, no interaction was found between the time the questionnaire was submitted and the year of study regarding the amount of exercise, sleep quality, and well-being, emphasizing that all students, regardless of what year of study they are in, showed better sleep quality and well-being and engaged in more exercise during the lockdown compared to the previous period. The study by Romero-Blanco et al [39] showed that first- and second-year students, but not final-year students, who increased the amount of physical activity during COVID-19 accounted for the difference in the exercise time by the fact that third-year students are required to take on a large number of written assignments, hence increasing their sitting time. In contrast, in this study, the undergraduate students of education had a similar curriculum during the first 3 years of studies; thus, no difference was found between the years of study with respect to physical activity.

Finally, an ordinal interaction was found between the time when the questionnaire was submitted and gender regarding the amount of exercise, that is, even though all students exercised more during the COVID-19 lockdown, male students engaged in a greater amount of exercise before and during the lockdown compared to female students, although female students had a more significant change during the COVID-19 lockdown. This is in line with other studies showing that male students, in general, engage in more sports and more physical activity because of the positive sensation they experience from exercise, stimulation, and enjoyment compared to female students [67]. In addition, male students mention enjoyment, challenges, social recognition, affiliation, competition, and strength as motivating factors for exercise, whereas female students mention preventing poor health and maintaining good health, weight management, and a pleasing appearance [68].

### Limitations

This study had several limitations. First, the study was conducted on a small and specific population most of whom are single and without children; therefore, our findings cannot be generalized to all undergraduate student populations, particularly those who are married and with children. Second, there was a difference between the students’ responses to the first and second questionnaires because the pre- COVID-19 questionnaire was given to the students by their teacher during 1 of their classes and they were given time to answer and submit during class. During the COVID-19 lockdown, all student-teacher communication was only online. Hence, the second questionnaire was sent via email, and not all students who answered the pre-COVID-19 lockdown questionnaire answered the second one. Although the number of respondents decreased, our findings were statistically significant. Third, it should be noted that there might be other factors that can affect well-being, sleep quality, and the amount of exercise that were not examined, such as health status, economic status, personality type, social status, and even living area (city vs rural locality). Finally, this effect was explored during the first lockdown only and lacks information regarding whether there could be a long-term, during-COVID-19 effect or whether the effect would similar during the second COVID-19 lockdown. This should be studied in continued research.

### Conclusion

In conclusion, although this study was conducted on a specific population of undergraduate students of education, and thus it is not possible to draw conclusions about the entire population, we showed a statistical correlation between the tested variables, indicating a beneficial effect on sleep quality, well-being, and amount of exercise in this population during the COVID-19 lockdown. These results can encourage people to understand that there is another positive aspect to crisis situations, such as lockdowns, and one can use such situations to improve the quality of life. The results can also inspire policymakers to propose programs focusing on promoting physical activity, and emphasizing sleep quality and well-being, to maintain a healthy lifestyle during lockdowns.

## Appendix

Multimedia Appendix 1 Working during the COVID-19 lockdown relative to normal days.

## References

[1] World Health Organization (2020). Statement on the second meeting of the International Health Regulations (2005) Emergency Committee regarding the outbreak of novel coronavirus (2019-nCoV). https://tinyurl.com/55f2kvmu.

[2] Gibney E (2020). Coronavirus lockdowns have changed the way Earth moves. Nature.

[3] Israel Coronavirus, 677 Cases and 3,286 Deaths. Worldometer.

[4] Rajkumar RP (2020). COVID-19 and mental health: a review of the existing literature. Asian J Psychiatr.

[5] Wynn R (2020). E-health in Norway before and during the initial phase of the Covid-19 pandemic. Stud Health Technol Inform.

[6] Roy S Insomnia Medication Prescriptions Increase with Spread of Coronavirus. Sleep Review.

[7] Mazza C, Ricci E, Biondi S, Colasanti M, Ferracuti S, Napoli C, Roma P (2020). A nationwide survey of psychological distress among Italian people during the COVID-19 pandemic: immediate psychological responses and associated factors. Int J Environ Res Public Health.

[8] Maugeri G, Castrogiovanni P, Battaglia G, Pippi R, D'Agata V, Palma A, Di Rosa M, Musumeci G (2020). The impact of physical activity on psychological health during Covid-19 pandemic in Italy. Heliyon.

[9] Yamada M, Kimura Y, Ishiyama D, Otobe Y, Suzuki M, Koyama S, Kikuchi T, Kusumi H, Arai H (2020). Effect of the COVID-19 epidemic on physical activity in community-dwelling older adults in Japan: a cross-sectional online survey. J Nutr Health Aging.

[10] Dinis J, Bragança M (2018). Quality of sleep and depression in college students: a systematic review. Sleep Sci.

[11] Stanton R, To QG, Khalesi S, Williams SL, Alley SJ, Thwaite TL, Fenning AS, Vandelanotte C (2020). Depression, anxiety and stress during COVID-19: associations with changes in physical activity, sleep, tobacco and alcohol use in Australian adults. Int J Environ Res Public Health.

[12] Duncan GE, Avery AR, Seto E, Tsang S (2020). Perceived change in physical activity levels and mental health during COVID-19: findings among adult twin pairs. PLoS One.

[13] Dabell J (2019). The importance of sleep. SecEd.

[14] Curcio G, Ferrara M, De Gennaro L (2006). Sleep loss, learning capacity and academic performance. Sleep Med Rev.

[15] Lau JTF, Yang X, Tsui HY, Pang E, Wing YK (2006). Positive mental health-related impacts of the SARS epidemic on the general public in Hong Kong and their associations with other negative impacts. J Infect.

[16] Javaheri S, Storfer-Isser A, Rosen CL, Redline S (2008). Sleep quality and elevated blood pressure in adolescents. Circulation.

[17] Litwiller B, Snyder LA, Taylor WD, Steele LM (2017). The relationship between sleep and work: a meta-analysis. J Appl Psychol.

[18] Pilcher JJ, Walters AS (1997). How sleep deprivation affects psychological variables related to college students' cognitive performance. J Am Coll Health.

[19] Abdulah DM, Musa DH (2020). Insomnia and stress of physicians during COVID-19 outbreak. Sleep Med X.

[20] Zhang C, Yang L, Liu S, Ma S, Wang Y, Cai Z, Du H, Li R, Kang L, Su M, Zhang J, Liu Z, Zhang B (2020). Survey of insomnia and related social psychological factors among medical staff involved in the 2019 novel coronavirus disease outbreak. Front Psychiatry.

[21] Orgilés M, Morales A, Delveccio E, Mazzeschi C, Espada JP (2020). Immediate psychological effects of COVID-19 quarantine in youth from Italy and Spain. SSRN J.

[22] Radek K, Burkes B, Levitt T, Baxter E, DiNardi A (2020). Global sleep health in a COVID-19 virus-infected world. Int Med.

[23] Warburton DER, Nicol CW, Bredin SSD (2006). Health benefits of physical activity: the evidence. CMAJ.

[24] Ancoli-Israel S (2001). "Sleep is not tangible" or what the Hebrew tradition has to say about sleep. Psychosom Med.

[25] Sherrill DL, Kotchou K, Quan SF (1998). Association of physical activity and human sleep disorders. Arch Intern Med.

[26] Morgenthaler T, Kramer M, Alessi C, Friedman L, Boehlecke B, Brown T, Coleman J, Kapur V, Lee-Chiong T, Owens J, Pancer J, Swick T, American Academy of Sleep Medicine (2006). Practice parameters for the psychological and behavioral treatment of insomnia: an update. An American academy of sleep medicine report. Sleep.

[27] Kredlow MA, Capozzoli MC, Hearon BA, Calkins AW, Otto MW (2015). The effects of physical activity on sleep: a meta-analytic review. J Behav Med.

[28] Kubitz KA, Landers DM, Petruzzello SJ, Han M (1996). The effects of acute and chronic exercise on sleep. A meta-analytic review. Sports Med.

[29] O’Connor PJ, Youngstedt SD (1995). Influence of exercise on human sleep. Exerc Sport Sci Rev.

[30] Youngstedt S, O'Connor PJ, Dishman R (1997). The effects of acute exercise on sleep: a quantitative synthesis. Sleep.

[31] Reid KJ, Baron KG, Lu B, Naylor E, Wolfe L, Zee PC (2010). Aerobic exercise improves self-reported sleep and quality of life in older adults with insomnia. Sleep Med.

[32] Nguyen-Michel ST, Unger JB, Hamilton J, Spruijt-Metz D (2006). Associations between physical activity and perceived stress/hassles in college students. Stress Health.

[33] Cooney G, Dwan K, Greig C (2013). Exercise for depression. Cochrane Database Syst Rev.

[34] Asmundson GJG, Fetzner MG, Deboer LB, Powers MB, Otto MW, Smits JAJ (2013). Let's get physical: a contemporary review of the anxiolytic effects of exercise for anxiety and its disorders. Depress Anxiety.

[35] Gorczynski P, Faulkner G (2010). Exercise therapy for schizophrenia. Cochrane Database Syst Rev.

[36] Luciano F, Cenacchi V, Vegro V, Pavei G (2021). COVID-19 lockdown: physical activity, sedentary behaviour and sleep in Italian medicine students. Eur J Sport Sci.

[37] Romero-Blanco C, Rodríguez-Almagro J, Onieva-Zafra MD, Parra-Fernández ML, Prado-Laguna MDC, Hernández-Martínez A (2020). Sleep pattern changes in nursing students during the COVID-19 lockdown. Int J Environ Res Public Health.

[38] Marelli S, Castelnuovo A, Somma A, Castronovo V, Mombelli S, Bottoni D, Leitner C, Fossati A, Ferini-Strambi L (2021). Impact of COVID-19 lockdown on sleep quality in university students and administration staff. J Neurol.

[39] Romero-Blanco C, Rodríguez-Almagro J, Onieva-Zafra MD, Parra-Fernández ML, Prado-Laguna MDC, Hernández-Martínez A (2020). Physical activity and sedentary lifestyle in university students: changes during confinement due to the COVID-19 pandemic. Int J Environ Res Public Health.

[40] Booth M (2000). Assessment of physical activity: an international perspective. Res Q Exerc Sport.

[41] Veit CT, Ware JE (1983). The structure of psychological distress and well-being in general populations. J Consult Clin Psychol.

[42] Florian V, Drori Y (1990). Mental Health Inventory (MHI): psychometric characteristics and normative data regarding the Israeli population. Psychologia Isr J Psychol.

[43] Zomer J, Peled R, Rubin A, Lavie P, Koella W, Ruther E, Schulz H (1985). Mini Sleep Questionnaire (MSQ) for screening large populations for EDS complaints in sleep. Sleep.

[44] Sinha M, Pande B, Sinha R (2020). Impact of COVID-19 lockdown on sleep-wake schedule and associated lifestyle related behavior: a national survey. J Public Health Res.

[45] Đogaš Z, Lušić Kalcina L, Pavlinac Dodig I, Demirović S, Madirazza K, Valić M, Pecotić R (2020). The effect of COVID-19 lockdown on lifestyle and mood in Croatian general population: a cross-sectional study. Croat Med J.

[46] Robinson E, Boyland E, Chisholm A, Harrold J, Maloney NG, Marty L, Mead BR, Noonan R, Hardman CA (2021). Obesity, eating behavior and physical activity during COVID-19 lockdown: a study of UK adults. Appetite.

[47] López-Bueno R, Calatayud J, Andersen LL, Balsalobre-Fernández C, Casaña J, Casajús JA, Smith L, López-Sánchez GF (2020). Immediate impact of the COVID-19 confinement on physical activity levels in Spanish adults. Sustainability.

[48] Sundarasen S, Chinna K, Kamaludin K, Nurunnabi M, Baloch GM, Khoshaim HB, Hossain SFA, Sukayt A (2020). Psychological impact of COVID-19 and lockdown among university students in Malaysia: implications and policy recommendations. Int J Environ Res Public Health.

[49] Son C, Hegde S, Smith A, Wang X, Sasangohar F (2020). Effects of COVID-19 on college students' mental health in the United States: interview survey study. J Med Internet Res.

[50] Altena E, Baglioni C, Espie CA, Ellis J, Gavriloff D, Holzinger B, Schlarb A, Frase L, Jernelöv Susanna, Riemann D (2020). Dealing with sleep problems during home confinement due to the COVID-19 outbreak: practical recommendations from a task force of the European CBT-I Academy. J Sleep Res.

[51] Di Stefano V, Battaglia G, Giustino V, Gagliardo A, D'Aleo M, Giannini O, Palma A, Brighina F (2021). Significant reduction of physical activity in patients with neuromuscular disease during COVID-19 pandemic: the long-term consequences of quarantine. J Neurol.

[52] Sandín B, Valiente RM, García-Escalera J, Chorot P (2020). Impacto psicológico de la pandemia de COVID-19: efectos negativos y positivos en población española asociados al periodo de confinamiento nacional. RPPC.

[53] Martínez-González MA, Varo JJ, Santos JL, De Irala J, Gibney M, Kearney J, Martínez JA (2001). Prevalence of physical activity during leisure time in the European Union. Med Sci Sports Exerc.

[54] Shigemura J, Ursano RJ, Morganstein JC, Kurosawa M, Benedek DM (2020). Public responses to the novel 2019 coronavirus (2019-nCoV) in Japan: mental health consequences and target populations. Psychiatry Clin Neurosci.

[55] Majumdar P, Biswas A, Sahu S (2020). COVID-19 pandemic and lockdown: cause of sleep disruption, depression, somatic pain, and increased screen exposure of office workers and students of India. Chronobiol Int.

[56] Brajša-Žganec A, Merkaš M, Šverko I (2010). Quality of life and leisure activities: how do leisure activities contribute to subjective well-being?. Soc Indic Res.

[57] Molina-García J, Castillo I, Queralt A (2011). Leisure-time physical activity and psychological well-being in university students. Psychol Rep.

[58] McMahon EM, Corcoran P, O'Regan G, Keeley H, Cannon M, Carli V, Wasserman C, Hadlaczky G, Sarchiapone M, Apter A, Balazs J, Balint M, Bobes J, Brunner R, Cozman D, Haring C, Iosue M, Kaess M, Kahn J, Nemes B, Podlogar T, Poštuvan V, Sáiz P, Sisask M, Tubiana A, Värnik P, Hoven CW, Wasserman D (2017). Physical activity in European adolescents and associations with anxiety, depression and well-being. Eur Child Adolesc Psychiatry.

[59] Haapasalo V, de Vries H, Vandelanotte C, Rosenkranz RR, Duncan MJ (2018). Cross-sectional associations between multiple lifestyle behaviours and excellent well-being in Australian adults. Prev Med.

[60] Steptoe A, Butler N (1996). Sports participation and emotional wellbeing in adolescents. Lancet.

[61] Ströhle A (2009). Physical activity, exercise, depression and anxiety disorders. J Neural Transm (Vienna).

[62] Wunsch K, Kasten N, Fuchs R (2017). The effect of physical activity on sleep quality, well-being, and affect in academic stress periods. NSS.

[63] Sullivan Bisson AN, Robinson SA, Lachman ME (2019). Walk to a better night of sleep: testing the relationship between physical activity and sleep. Sleep Health.

[64] Lund HG, Reider BD, Whiting AB, Prichard JR (2010). Sleep patterns and predictors of disturbed sleep in a large population of college students. J Adolesc Health.

[65] Taylor DJ, Bramoweth AD (2010). Patterns and consequences of inadequate sleep in college students: substance use and motor vehicle accidents. J Adolesc Health.

[66] Taylor DJ, Bramoweth AD, Grieser EA, Tatum JI, Roane BM (2013). Epidemiology of insomnia in college students: relationship with mental health, quality of life, and substance use difficulties. Behav Ther.

[67] Lauderdale ME, Yli-Piipari S, Irwin CC, Layne TE (2015). Gender differences regarding motivation for physical activity among college students: a self-determination approach. TPE.

[68] Gao Z, Xiang P (2008). College students' motivation toward weight training: an application of expectancy-value model. J Teach Phys Educ.

