# Supplementary material for: The Beneficial Effect of the First COVID-19 Lockdown on Undergraduate Students of Education: Prospective Cohort Study
Source: JMIR Form Res. 2022 Feb 23;6(2):e27286. doi: 10.2196/27286 (PMC8869364; doi:10.2196/27286)
Supplement: Multimedia Appendix 1 [file formative_v6i2e27286_app1.docx]

**Table S1.** Working during the COVID-19 lockdown relative to normal days (N=164).

| Work status | n (%) |
| --- | --- |
| Did not work at all | 62 (37.8) |
| Worked less than normal times | 82 (50.0) |
| Worked normal times | 12 (7.3) |
| Worked more or much more than normal times | 8 (4.9) |
